# A Method for Workout Video Classification via Explainable and Federated Learning

**DOI:** 10.3390/bioengineering13060603

**Published:** 2026-05-22

**Authors:** Ludovica Ciardiello, Patrizia Agnello, Marta Petyx, Fabio Martinelli, Mario Cesarelli, Antonella Santone, Francesco Mercaldo

**Affiliations:** 1 Department of Medicine and Health Sciences “Vincenzo Tiberio”, University of Molise, 86100 Campobasso, Italy; antonella.santone@unimol.it; 2 Istituto Nazionale per l’Assicurazione contro gli Infortuni sul Lavoro, 00144 Rome, Italy; p.agnello@inail.it (P.A.); m.petyx@inail.it (M.P.); 3 Institute for High Performance Computing and Networking, National Research Council of Italy (CNR), 87036 Rende, Italy; fabio.martinelli@icar.cnr.it; 4 Department of Engineering, University of Sannio, 82100 Benevento, Italy; mcesarelli@unisannio.it

**Keywords:** workout, classification, federated machine learning, explainability

## Abstract

In recent years, the widespread availability of wearable devices and smartphones has enabled the large-scale collection of human activity data, fostering new opportunities for automatic workout recognition and personalized fitness monitoring. However, the centralized storage of video recordings raises critical privacy concerns, particularly when raw data contain identifiable individuals. Federated Machine Learning provides a paradigm designed with the aim of reducing privacy risks; here, models are collaboratively trained across distributed clients without sharing their sensitive data. In this paper, we propose an approach for workout video classification with Federated Machine Learning, enhanced by explainability through Gradient-weighted Class-Activation Mapping. The proposed method is evaluated on a real-world multi-class exercise video dataset, organized into eight biomechanically coherent macro-classes. In the experimental analysis, we consider several federated configurations in terms of the number of clients, the chosen aggregation strategy, and global communication rounds. The obtained results demonstrate that different aggregation strategies achieve comparable overall accuracy, while explainability effectively highlights the discriminative regions associated with exercise execution, revealing meaningful differences in model behavior between aggregation strategies and uncovering misclassifications driven by contextual biases, demonstrating the trustworthiness of the proposed approach for explainable workout video classification.

## 1. Introduction

The recent proliferation of wearable devices, smartphones, and cameras has enabled the large-scale acquisition of human activity data, paving the way for automatic exercise recognition and personalized fitness monitoring [1]. However, the centralized collection of video data raises serious privacy concerns, since raw recordings may contain sensitive personal information and identifiable individuals. This has motivated the development of decentralized learning paradigms that maintain user privacy while enabling collaborative model training across distributed devices.

Federated Machine Learning (FML) is a privacy-preserving machine learning paradigm in which clients collaboratively train a shared global model while retaining their data locally, thus avoiding the direct transmission of their sensitive information to a central server. The approach introduced by McMahan et al. [2] aggregates locally computed model updates via weighted averaging, effectively enabling scalable federated optimization without raw data exchange. Nonetheless, challenges such as statistical heterogeneity, communication overheads, and convergence on non-independent and identically distributed (non-IID) data remain open research issues in Federated Machine Learning [3].

In parallel, deep learning models for object detection and human action recognition have achieved really interesting performance in both accuracy and speed, enabling real-time detection [4,5]. In this direction, the “You Only Look Once” (YOLO) model family of single-stage object detectors offers real-time detection capabilities that are applicable to video and activity analysis tasks, with the more recent YOLO architectures further improving accuracy–speed trade-offs and supporting multiple vision tasks [6]. Despite their effectiveness, deep neural networks often lack transparency, operating as black-box models that limit interpretability in safety-critical applications [7]. Explainability techniques such as Gradient-weighted Class-Activation Mapping (Grad-CAM) provide visual explanations by highlighting the image regions that influence model decisions most strongly, enabling qualitative analyses of model behavior to be conducted [8].

Starting from these considerations, in this paper, we propose a Federated Machine Learning method for privacy-preserving workout video classification enhanced by explainability through Grad-CAM.

Below, we itemize the main contributions of the proposed method:

- We design an FML method by exploiting the YOLO model for distributed training on non-IID workout videos across multiple clients.
- We systematically explore different aggregation strategies (i.e., FedAvg and FedProx) and analyze their effectiveness with respect to class imbalance and heterogeneous client data [3].
- We enhance model explainability by applying two different explainability techniques, i.e., Grad-CAM and Grad-CAM++, with the aim of visualizing the discriminative regions used by the model to classify workout video frames, ultimately improving the trustworthiness of model predictions.
- We consider a real-world multi-class exercise video dataset, including macro-class groupings and augmentation to reduce subject-specific biases in learning.

The paper proceeds as follows: In the next section, we review the current state-of-the-art literature related to human activity recognition, FML and deep learning explainability. In Section 3, we introduce the method we designed and developed for privacy-preserving and explainable workout video classification. The experimental analysis is presented in Section 4 with the aim of demonstrating the effectiveness of the proposed method. Finally, in the last section, the conclusions are presented and future research directions are discussed.

## 2. Related Work

Human activity recognition (HAR) aims to automatically identify human actions from data modalities such as video or sensors, with applications in healthcare, sports analytics, and human–computer interactions. Early methods relied on handcrafted features such as optical flow and pose estimations, but convolutional neural networks and Transformer-based models have substantially raised recognition performance by learning spatiotemporal representations directly [9]. Real-time object-detection frameworks such as the YOLO family balance speed and accuracy, making them suitable for video activity recognition in edge and federated settings. YOLOv8 introduces architecture improvements that enhance detection precision and versatility across tasks including object detection and pose estimation [6].

Federated Machine Learning enables collaborative model training on decentralized data; this is critical when centralized data collection is not feasible due to privacy or regulatory constraints [10]. The Federated Averaging (FedAvg) algorithm [11] aggregates local model updates from clients into a global model and has been widely applied to distributed image and sensor data tasks [2]. To better handle the statistical heterogeneity that is inherent in non-IID settings (e.g., varied exercise execution styles or subject distributions), FedProx introduces a proximal term to stabilize local updates and reduce client drift [3]. More recent works have explored advanced strategies to further improve performance and privacy in federated settings. For instance, fine-tuning and aggregation mechanisms have been proposed to enhance model adaptation, as in FedFTHA, which combines local fine-tuning with head aggregation to improve robustness and accuracy across clients [12]. Furthermore, recent studies investigate the trade-off between local personalization and global generalization, proposing methods that jointly optimize both aspects to better handle heterogeneous client data distributions [13]. In addition, privacy-preserving recommender and learning systems have gained increasing attention. A comprehensive survey highlights the evolution from conventional defense mechanisms toward decentralized approaches such as Federated Machine Learning and blockchain-based solutions [14]. Recent surveys highlight the growing interest in applying FML to human activity recognition, particularly for wearable and mobile devices where privacy is paramount [15].

In the work by Kalabakov et al. [16], human activity recognition is addressed using both Federated Machine Learning and centralized learning based on data collected from wearable devices, such as inertial sensors. This approach requires users to wear dedicated devices for data collection. Gad et al. [17] instead propose FedAKD, a Federated Machine Learning method that integrates knowledge distillation to improve model heterogeneity handling and communication efficiency. Furthermore, Iacob et al. [18] studied Federated Machine Learning in multimodal human activity recognition scenarios, analyzing the impact of different privacy levels and the joint use of multiple sensors. While these approaches demonstrate the effectiveness of Federated Machine Learning in sensor-based HAR settings, they primarily rely on wearable or multimodal sensor data. However, video-based HAR in a federated context remains under-explored compared to sensor-based applications.

In the survey by Alomar et al. [19] and in the work [20], the evolution of models used for human activity recognition is summarized, starting from CNNs and RNNs and progressing to Vision Transformers, which have improved the ability to model both spatial and temporal dependencies. Recent advances in video-based human action recognition have introduced efficient Transformer architectures such as the Video Swin Transformer [21], which incorporates locality-aware self-attention to improve computational efficiency while achieving state-of-the-art performance on large-scale benchmarks. However, in contrast to the aforementioned works, our approach integrates a Federated Machine Learning setting and introduces an explainability analysis in order to better understand the model’s behavior and improve the interpretability of its decisions.

The explainability of deep vision models is especially important when these models are deployed in health and fitness applications, where end-user trust and diagnostic transparency are essential. Grad-CAM generates visual explanations by backpropagating gradients to the final convolutional layer to produce class-discriminative heatmaps, which are useful for understanding what image regions drive decisions [8]. While they are widely used in image categorization, Grad-CAM and related methods are increasingly adopted in action recognition to verify that models attend to meaningful motion patterns rather than spurious background cues [22]. Integrating such methods with federated training can help diagnose biases introduced by non-IID data distributions across clients.

The existing literature addresses Federated Machine Learning, explainable AI, and activity recognition, but these are primarily discussed in isolation or in relation to sensor-based HAR. To the best of our knowledge, this work is among the first to combine federated YOLOv8 training with explainability via Grad-CAM in a distributed, video-based exercise recognition setting, bridging privacy, performance, and interpretability concerns.

## 3. Method

**Figure 1:**
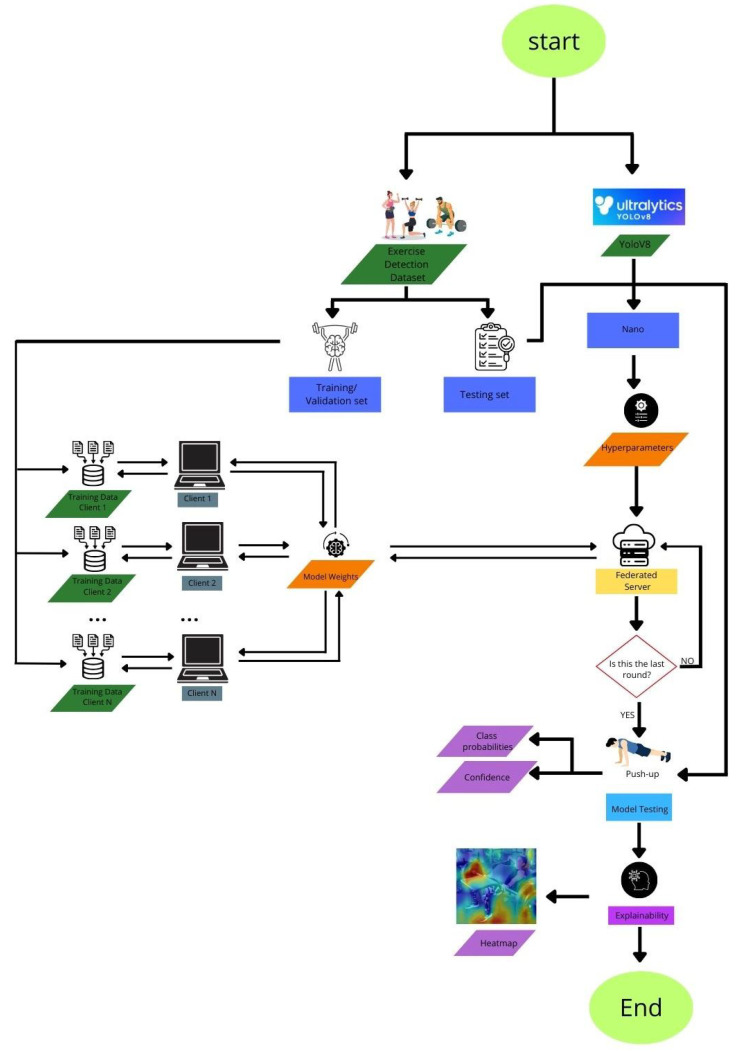
Workflow of the proposed method for workout video classification, combining Federated Machine Learning and explainable classification via Grad-CAM.

In this section, we present the method we designed and developed for privacy-preserving workout video classification based on Federated Machine Learning, with explainability through Grad-CAM. The workflow of the proposed method, shown in Figure 1, exploits a dataset which is organized into different folders corresponding to the considered classes, namely: floor_exercise, bench_exercise, standing_lift, biceps_curl, shoulder_exercise, hanging_exercise, machine_exercise and tricep_pushdown. The dataset is then split into three subsets: training, used for model training; validation, employed to monitor performance during learning; and test, reserved for final evaluation. Cross-validation was not employed in this work; instead, a fixed train/validation/test split was used to ensure a consistent and efficient evaluation protocol.

The dataset is subsequently distributed among different clients, assigning each a portion of the training and validation sets to ensure proper data localization and simulate realistic distributed learning scenarios. The scenario under consideration involves the recognition of physical exercises, where each client has data corresponding to different exercise classes. For local training, the YOLOv8-CLS model is employed in a frame-level classification setting. The model is optimized using the Cross-Entropy Loss, which is commonly employed for multi-class classification tasks. This loss function measures the discrepancy between the predicted class probabilities and the ground truth labels, encouraging the model to assign high probability to the correct class while penalizing incorrect predictions. To maintain stability and consistency in the federated process, key hyperparameters, i.e., learning rate, batch size, and number of local training epochs, are kept uniform across all clients. Similarly, the aggregation algorithm used by the central server (e.g., FedAvg or FedProx) is applied consistently in each federated round. In each round, clients train the model locally on their own data and send the updated weights to the federated server. The server aggregates the weights according to a defined fusion strategy and redistributes the updated global model to the clients. This cycle repeats for a predetermined number of rounds or until a defined stopping criterion is reached.

At the end of training, the global model undergoes model testing using the test set, comprising both images and videos. Performance is analyzed through the model output, including the predicted class and class probability.

To enhance model transparency and interpretability, an explainability module based on CAM techniques is applied. Specifically, Grad-CAM and Grad-CAM++ are used to generate heatmaps highlighting the image regions most influential in the model’s decision-making process. These visual representations provide insight into the rationale behind the predictions, contributing to a more interpretable and reliable system. In fact, Grad-CAM generates visual explanations by computing the gradient of a target class score with respect to the feature maps of a convolutional layer. It produces coarse, class-specific heatmaps that highlight regions most influential for the model’s prediction. Grad-CAM uses global average pooling of the gradients, resulting in a single importance weight per feature map; while this is effective, it tends to produce broad activation regions and can struggle in scenarios with multiple discriminative instances in the same image.

Differently, Grad-CAM++, an extension of Grad-CAM, refines the weighting mechanism by incorporating pixel-wise gradient importance through higher-order derivatives. This allows it to assign different levels of importance to individual spatial locations within each feature map. As a result, Grad-CAM++ provides sharper, more localized heatmaps and performs better when multiple object instances contribute to the prediction. It is particularly advantageous for detecting fine-grained cues and for explaining models in dense or cluttered scenes.

## 4. Results

In this section, we present the real-world dataset we exploited to show the effectiveness of the proposed method. As a matter of fact, we exploited a dataset composed from workout videos available on the Kaggle website [23].

We considered the videos contained in the verified_data folder, which includes preprocessed, short and noise-free clips lasting 10–13 s each.

**Figure 2:**
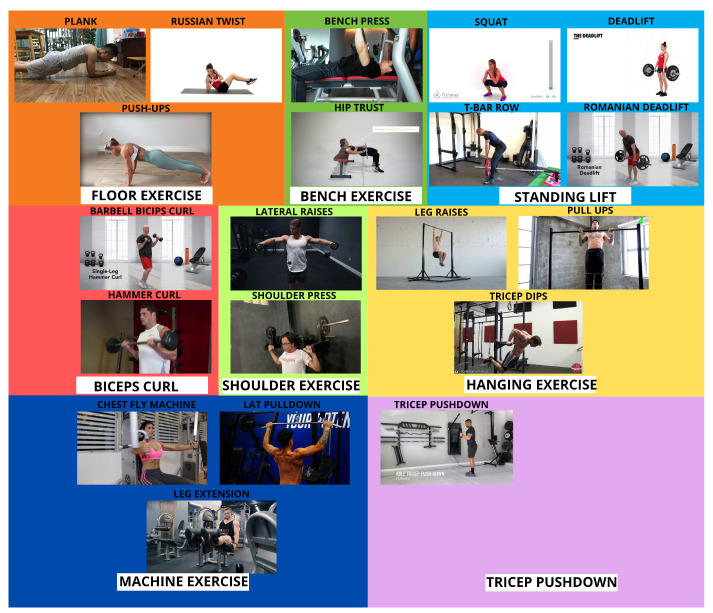
Class reorganization process showing how the original exercise categories were grouped into the eight target classes used for classification.

We considered eight classes, as previously introduced. Figure 2 shows several examples of images extracted from the dataset videos. The eight class groupings were based on biomechanical and functional similarity, combining exercises that involve similar muscle groups or share analogous movements. Here, we describe the classes in detail:

- Class 1: floor_exercise (highlighted in Figure 2 in orange), including plank, push-ups and Russian twist, which are all floor-based exercises primarily targeting core strengthening.
- Class 2: bench_exercise (highlighted in Figure 2 in dark green), including bench press, incline bench press, decline bench press and hip thrust, mainly involving chest, shoulders and glutes, which are often performed on a bench or support.
- Class 3: standing_lift (highlighted in Figure 2 in light blue), including squat, deadlift, Romanian deadlift and T-bar row, which are performed standing and which target the legs, back and core, involving lifting and pushing movements.
- Class 4: biceps_curl (highlighted in Figure 2 in red), including barbell biceps curl and hammer curl, which are isolated exercises for bicep strengthening.
- Class 5: shoulder_exercise (highlighted in Figure 2 in light green), including shoulder press and lateral raise, which are exercises targeting the shoulder muscles.
- Class 6: hanging_exercise (highlighted in Figure 2 in yellow), including pull-up, tricep dips and leg raises, which are performed by hanging or supported by bars, focusing on the back, triceps and abdominal muscles.
- Class 7: machine_exercise (highlighted in Figure 2 in blue), including the chest fly machine, leg extension and lat pull-down, which are performed using gym machines that guide the movement.
- Class 8: tricep_pushdown (highlighted in Figure 2 in purple), which is an isolated exercise targeting the triceps.

The dataset comprises the following number of images for each class: floor_exercise (2129 images), bench_exercise (1879 images), standing_lift (1999 images), biceps_curl (1020 images), shoulder_exercise (718 images), hanging_exercise (1412 images), machine_exercise (1299 images) and tricep_pushdown (278 images).

With the aim of improving model accuracy and to avoid overfitting, a data-augmentation pipeline was applied, consisting of the following techniques:

- Horizontal flip: with a 50% probability, images were flipped left-to-right, which is useful for symmetric exercises.
- Shift, scale, and rotate: images can be translated up to 20% in any direction, scaled up to 30%, and rotated ±45°, introducing variability in position and perspective.
- Color jitter: random changes in brightness, contrast, saturation and hue, enhancing robustness to varying lighting conditions.
- Coarse dropout: random rectangular regions are blacked out or removed (up to 10 per frame), forcing the model to focus on general exercise features.
- Gaussian blur: with a 30% probability, images are slightly blurred, simulating fast movements or low-quality videos.

After applying the data-augmentation pipeline, the dataset consists of the following number of images per class: floor_exercise (6387 images), bench_exercise (5655 images), standing_lift (5997 images), biceps_curl (3060 images), shoulder_exercise (2154 images), hanging_exercise (3921 images), machine_exercise (3897 images) and tricep_pushdown (834 images).

Subsequently, the videos were converted into frames and split into training and validation sets, distributing the data among the different clients. Particular care was taken to avoid placing videos of the same individual in different clients or sets, thereby preventing data leakage. As a result, the training is non-i.i.d. (non-identically distributed), with a variable number of images per class across different clients.

Table 1 reports the exact number of images per class for each client in the training and validation sets, highlighting the non-uniform distribution of data across the different clients. The test dataset, on the other hand, consists of 595 images and is identical for all clients, ensuring a consistent and comparable evaluation of the model’s performance.

A series of experiments was conducted, as reported in Table 2, in which the several hyperparameters were systematically varied for each experiment. Specifically, we considered the number of clients, defined as the number of devices participating in the Federated Machine Learning process; the number of local training epochs, corresponding to the number of training iterations performed by each client on its local dataset; the number of global rounds, representing the communication cycles between the central server and the clients; and the aggregation strategy, used by the server to combine the locally updated model parameters.

As reported in Table 2, the experiments were conducted with two, three and four clients. Three and five global aggregation rounds were used, combined with the FedAvg and FedProx strategies. The number of local training epochs was kept fixed at eight for all experiments. For optimization, the stochastic gradient descent (SGD) was used for FedAvg, while proximal SGD was employed for FedProx to account for the regularization term introduced in the algorithm. All experiments were conducted using a fixed random seed (1312) to ensure reproducibility of the results. For FedProx, the proximal term coefficient was set to $1\times 10^{-4}$ to regularize local updates and mitigate the effects of data heterogeneity in non-IID settings.

**Figure 3:**
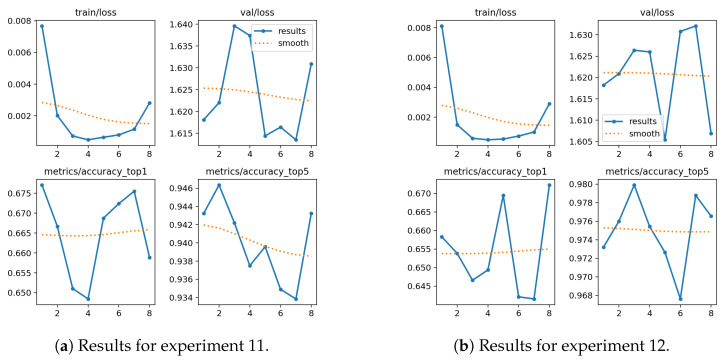
The best experimental results.

Analyzing the results reported in Table 3, we focus on the best-performing experiments involving four clients (Experiments 11 and 12, shown in Figure 3a,b). This selection allows for a meaningful evaluation of the impact of different aggregation strategies (FedAvg and FedProx) on model accuracy.

From the loss analysis, both approaches exhibit similar trends: the training loss rapidly decreases toward zero, reaching values between 0.0005 and 0.002, indicating effective learning during the training phase. The validation loss reaches a minimum of 1.615 for FedAvg, while FedProx achieves a slightly lower value of 1.605, showing only a marginal difference and a slight advantage of the latter in controlling the error.

Considering top-1 accuracy, the two models perform almost identically: FedAvg achieves a maximum value of 0.675, closely followed by FedProx at 0.672. A more noticeable difference emerges in terms of top-5 accuracy, where both approaches show a substantial improvement, reaching 0.946 for FedAvg and 0.980 for FedProx. This indicates that, even when the model fails to predict the correct class as its top choice, the correct label is almost always included among the top five predictions.

**Figure 4:**
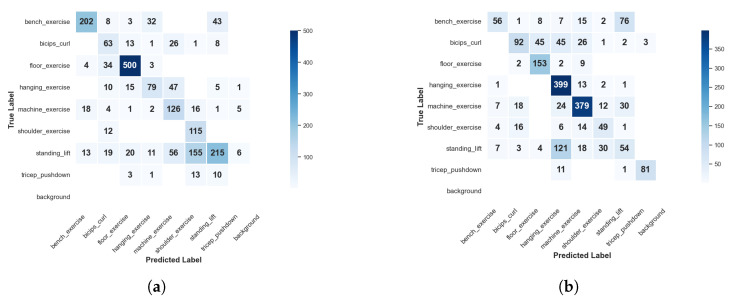
The confusion matrices corresponding to experiments 11 and 12, i.e., the ones obtaining the best accuracy. (**a**) The confusion matrix corresponding to experiment 11. (**b**) The confusion matrix corresponding to experiment 12.

Although FedAvg and FedProx achieve comparable overall performance, the two aggregation strategies implicitly optimize different objectives. An analysis of the confusion matrices Figure 4 reveals distinct behavioral patterns. Specifically, FedAvg (Figure 4a) tends to favor classes that are over-represented in dominant clients, resulting in models that are highly specialized but imbalanced. The FedAvg (Figure 4b) based model accurately recognizes the floor_exercise class with 500 correct predictions and performs well on standing_lift (215 correct) and bench_exercise (202 correct). However, it struggles to correctly classify hanging_exercise (79 correct) and machine_exercise (126 correct), frequently confusing them with other categories.

In contrast, FedProx mitigates the impact of non-IID local data distributions, improving the recognition of underrepresented classes at the cost of reduced performance on dominant ones. Notably, the FedProx based model excels in identifying machine_exercise with 379 correct predictions (compared to 126 for FedAvg) and hanging_exercise with 339 correct classifications (versus only 79 in the FedAvg case). However, this improvement comes at the expense of performance on floor_exercise (152 correct predictions) and standing_lift, where only 54 samples are correctly classified.

### 4.1. Comparison with Centralized Baseline

To provide a centralized baseline, we merged all the data from the different clients into a single dataset. The resulting data distribution is reported in Table 4.

**Figure 5:**
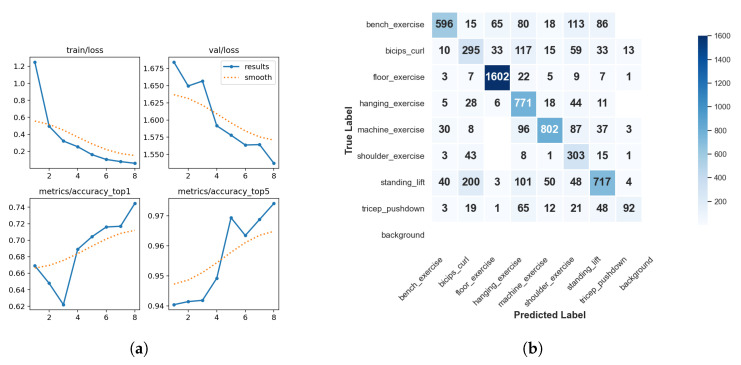
(**a**) Results of the centralized model. (**b**) Confusion matrix of the centralized model.

Using the same hyperparameters as in the federated setting and training for eight epochs, we obtained the results shown in Figure 5a.

As illustrated in the figure, the training loss decreases smoothly and rapidly from approximately 1.2 to 0.1. A similar trend is observed for the validation loss, indicating good generalization without evident overfitting. In terms of accuracy, the top-1 metric reaches approximately 74%, while the top-5 accuracy exceeds 97%.

The analysis of the confusion matrices Figure 5b confirms the results observed in the quantitative metrics. The centralized model exhibits a more pronounced diagonal, indicating a higher classification accuracy. In contrast, the federated model shows greater dispersion of values outside the diagonal, reflecting increased uncertainty, particularly among classes with similar characteristics. Despite the centralized approach achieving higher numerical performance, the federated setting remains competitive, especially considering the challenges introduced by data decentralization and non-IID distributions across clients. This highlights the effectiveness of Federated Machine Learning in maintaining reasonable performance while preserving data locality.

**Figure 6:**
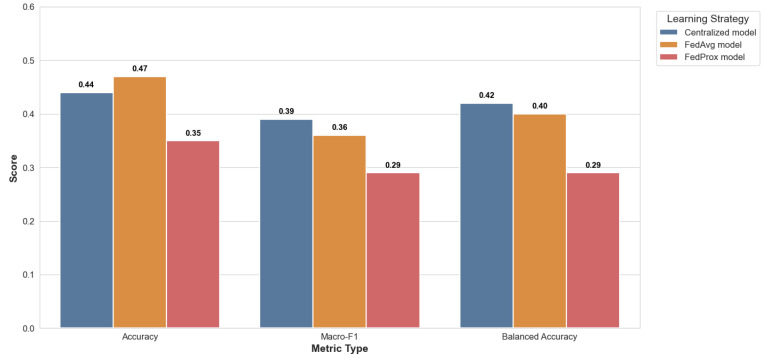
Comparison of global performance metrics.

The results in Figure 6 show that FedAvg achieves the highest accuracy (0.47), highlighting the ability of the federated model to obtain competitive performance even without centralized access to the data. However, the gap between accuracy and balanced metrics indicates a greater sensitivity to majority classes, consistent with the distributed and non-IID nature of the problem. The centralized model, while representing a higher reference in terms of overall dataset balance, achieves comparable performance (accuracy, 0.44; Macro-F1, 0.39; balanced accuracy, 0.42). FedProx, instead, shows lower performance (accuracy, 0.35) while remaining a useful approach for handling data heterogeneity. Overall, the results show that the federated approach, in particular FedAvg, achieves competitive performance compared to the centralized model, while preserving the advantages related to privacy constraints and the distributed nature of the data.

### 4.2. Explainability Analysis

With regard to explainability analysis, we employed the models corresponding to experiments 11 and 12, as reported in Table 2. The analysis focuses on two representative classes, namely floor_exercise and machine_exercise, which were selected because they achieved the best classification performance, as indicated by the confusion matrix.

Each figure includes the original input frame alongside the corresponding Grad-CAM and Grad-CAM++ visualizations. Above each activation map, the top-3 predicted classes and their associated confidence scores are reported. The color scale ranges from warm colors (red and yellow), highlighting image regions that contribute most strongly to the model’s prediction, to cool colors (purple and blue), which indicate areas with lower relevance to the classification outcome.

When analyzing the first class, floor_exercise, it can be observed that, in the first image, both models (experiment 11 and experiment 12) correctly classify the action with a confidence of 100%. However, the CAMs reveal notable differences in the decision-making processes of the two models.

**Figure 7:**
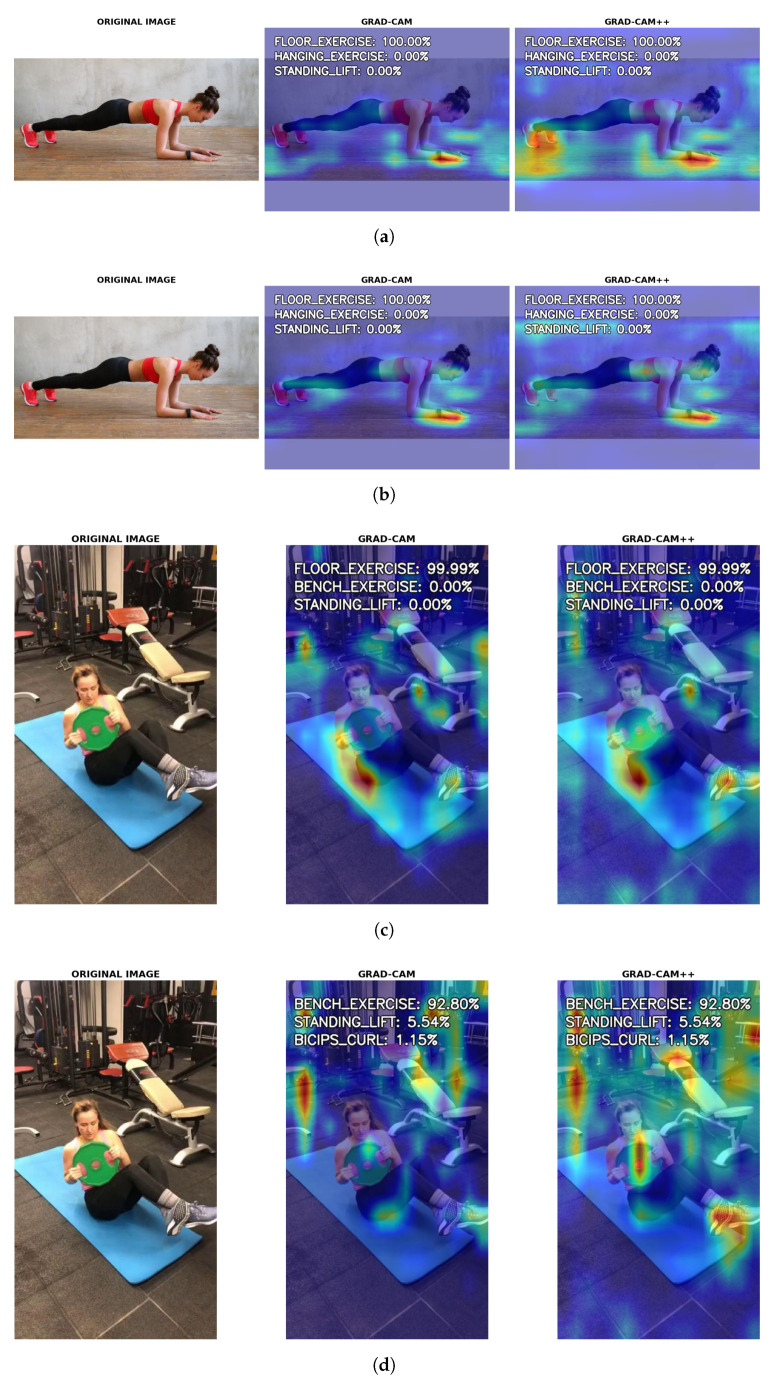
CAMs illustrating the model’s attention for the floor_exercise class predictions. (**a**) Experiment 11, plank exercise; (**b**) Experiment 12, plank exercise; (**c**) Experiment 11, russian twist exercise; (**d**) Experiment 12, russian twist exercise.

In the case of experiment 11 (Figure 7a), the Grad-CAM primarily focuses on the contact areas between the forearms and the floor, also showing a slight diffuse activation over the ground surface. In contrast, Grad-CAM++ highlights stronger activation both at the contact points between the feet and the floor and between the forearms and the ground. For experiment 12 (Figure 7b), the Grad-CAM and Grad-CAM++ maps appear more similar to each other and mainly concentrate on the region where the forearm is in contact with the floor, with a slight additional activation in the head area. In both cases, the models have correctly learned that, for a floor_exercise—specifically, the plank—the most discriminative visual features are the body’s contact points with the floor, particularly the feet and forearms.

The third and fourth images illustrate a representative case in which the model from Experiment 11 successfully classifies the exercise, whereas the model from Experiment 12 fails, in agreement with the corresponding confusion matrix.

In the third image (Figure 7c), associated with the correct prediction, both CAMs mainly focus on the subject’s body, particularly on the pelvic region (the main point of contact with the ground) and the shoes, while Grad-CAM++ also shows a slight activation in the torso area. Conversely, in the fourth image (Figure 7d), the model from Experiment 12 incorrectly classifies the exercise as bench_exercise. The corresponding CAMs reveal a strong activation on an empty weight bench in the background, indicating that the model was influenced by contextual elements rather than by the biomechanical characteristics of the exercise.

Analyzing the second class, machine_exercise, it is observed that the model from experiment 12 correctly identifies the exercise, whereas the model from experiment 11 makes mistakes.

**Figure 8:**
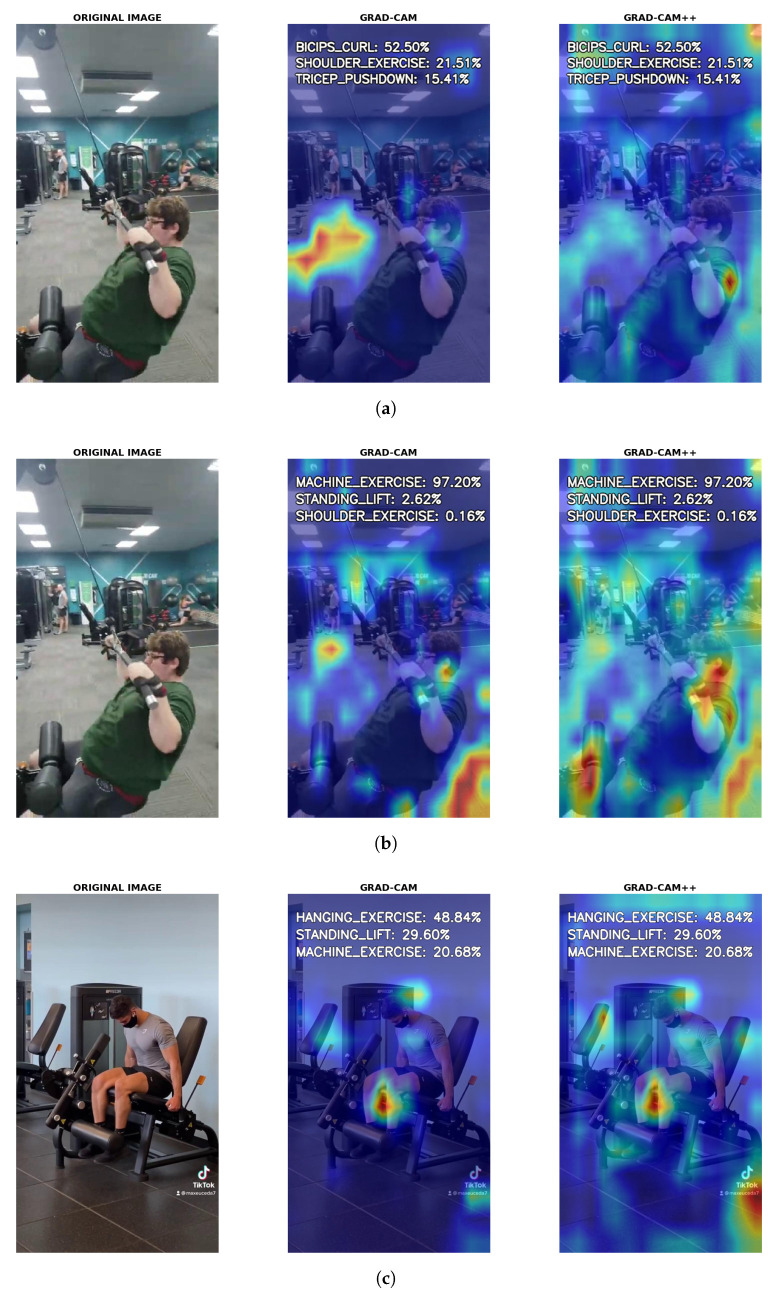

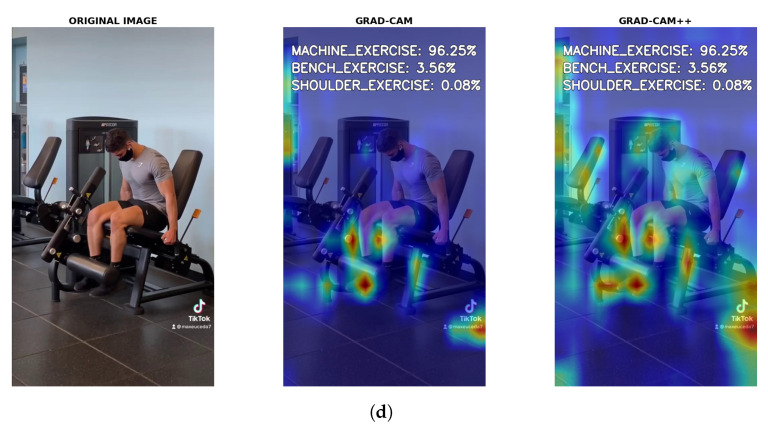
CAMs illustrating the model’s attention for the machine_exercise class predictions. (**a**) Experiment 11, lat pulldown exercise; (**b**) Experiment 12, lat pulldown exercise; (**c**) Experiment 11, leg extension exercise; (**d**) Experiment 12, leg extension exercise.

In the first image (Figure 8a), the experiment 11 model classifies the action as biceps_curl with a confidence of 58.58% and the correct category does not appear in the top-3 predictions. The Grad-CAM highlights mainly the floor, while Grad-CAM++ focuses on the man’s left arm. Isolating the arm movement, the flexion closely resembles a biceps curl, leading the model to misclassify the exercise.

The second image (Figure 8b) shows CAMs with stronger activation, concentrated on the structure of the machine, the cables, and the bar, as well as on the man’s posture, indicating a better recognition of the exercise context.

In the third image (Figure 8c), the model misclassifies the exercise as hanging_exercise, although the correct category (machine_exercise) appears in the top-3 with a probability of 18.33%. The CAMs show hot regions concentrated on the knees and partially on the head.

In the fourth image (Figure 8d), the Grad-CAM highlights mainly the machine structure, while Grad-CAM++ shows activation not only on the knees but also on the shins, suggesting that the model considers both the body and the machine for correctly classifying the exercise.

### 4.3. Ablation Test

We conducted two ablation studies. In the first, we segmented the clothing present in the images and applied masks to modify their color, with the aim of evaluating whether clothing influences the model’s decisions.

**Figure 9:**
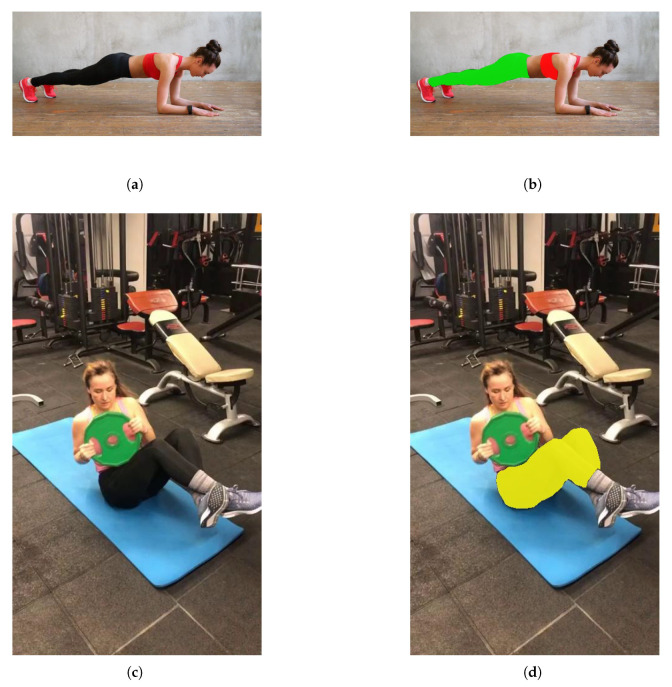
Comparison between the original image and the image with a mask applied to the clothing. (**a**) Original plank image; (**b**) Plank image with mask; (**c**) Original russian twist image; (**d**) Russian twist image with mask.

For the floor exercise class, in Figure 9a,b, we changed the color of the trousers to green and the top to red, while in Figure 9c,d, we changed the trousers to yellow. Grad-CAM was then used to analyze the regions on which the model focuses its attention.

**Figure 10:**
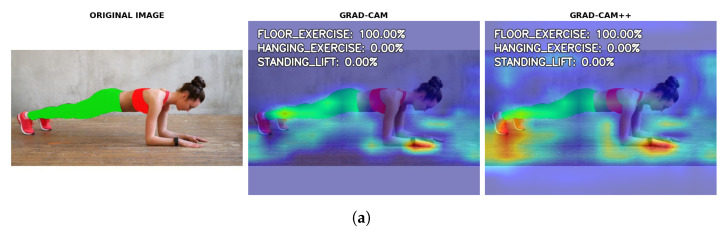

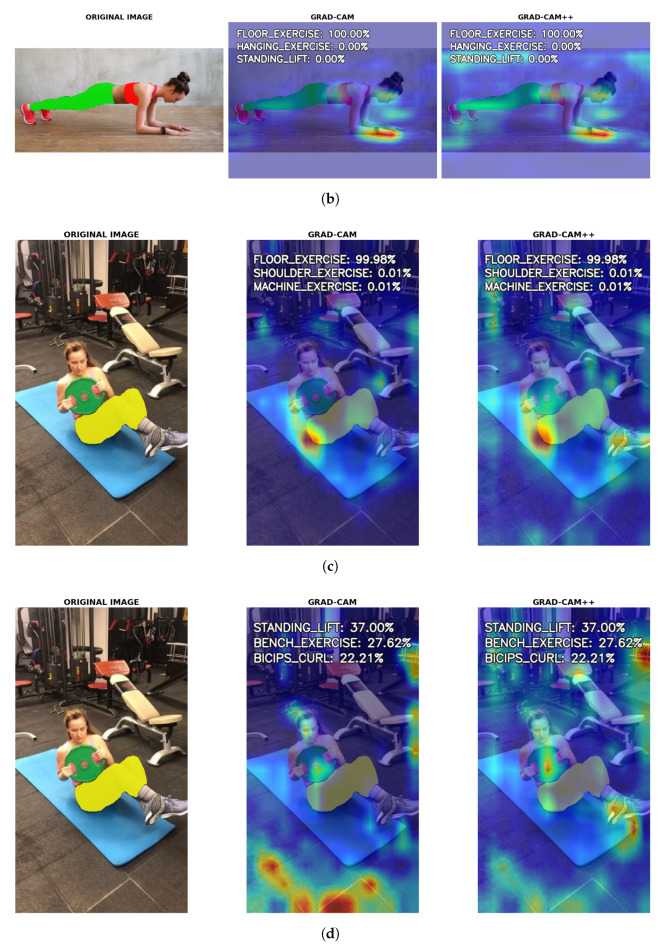
CAMs illustrating the model’s attention for the floor exercise class predictions with mask applied. (**a**) Experiment 11, plank exercise; (**b**) Experiment 12, plank exercise; (**c**) Experiment 11, russian twist exercise; (**d**) Experiment 12, russian twist exercise.

In Figure 10, the comparison with the original Grad-CAM maps does not show significant differences in either the predictions or the activation maps, for both experiment 11 (Figure 10a) and experiment 12 (Figure 10b) models. In contrast, in Figure 10, the Grad-CAM for experiment 11 (Figure 10c) remains similar to the original, whereas for experiment 12 (Figure 10d), the classification remains incorrect (consistent with the original result), but a shift in the attention regions is observed, with increased focus on the foreground floor.

**Figure 11:**
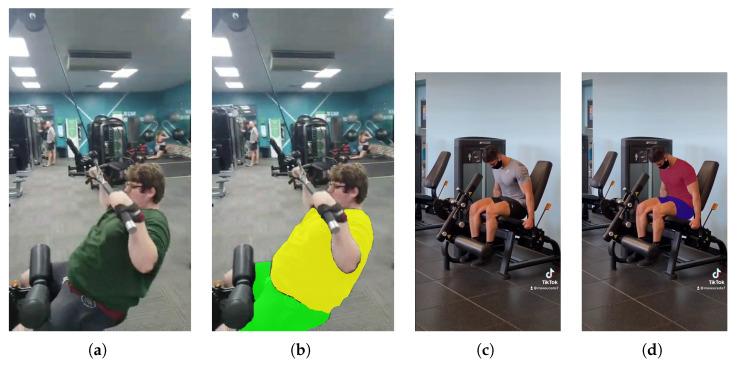
Comparison between the original image and the image with a mask applied to the clothing. (**a**) Original lat pulldown image; (**b**) Lat pulldown image with mask; (**c**) Original leg extension image; (**d**) Leg extension image with mask.

For the machine exercise class, in Figure 11a,b, we changed the shirt color to yellow and the trousers to green, while in Figure 11c,d, the shirt was changed to red and the trousers to blue.

**Figure 12:**
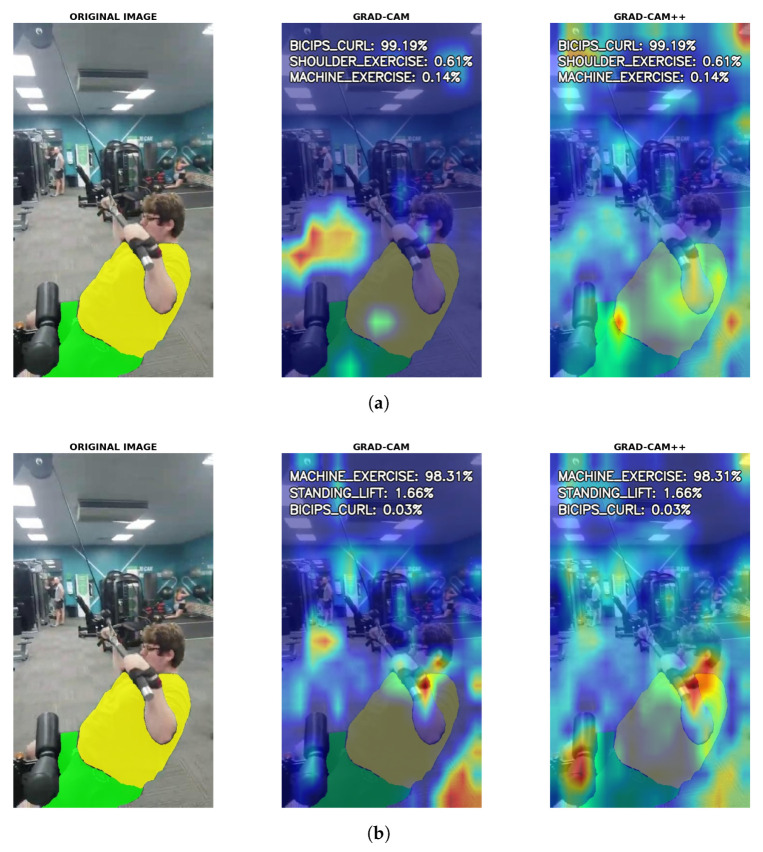

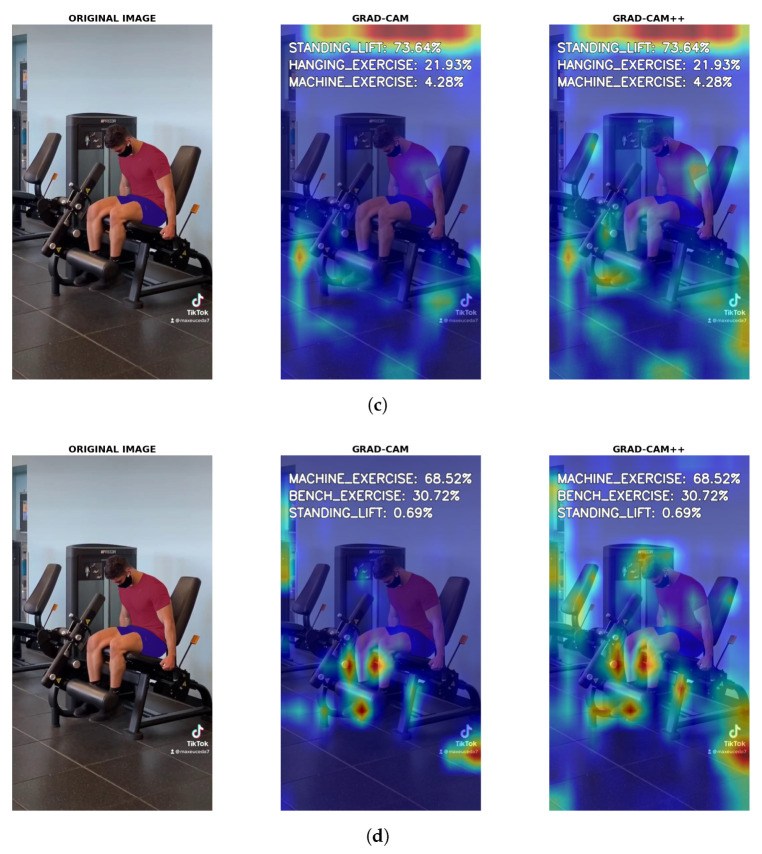
CAMs illustrating the model’s attention for the machine exercise class predictions with mask applied. (**a**) Experiment 11, lat pulldown exercise; (**b**) Experiment 12, lat pulldown exercise; (**c**) Experiment 11, leg extension exercise; (**d**) Experiment 12, leg extension exercise.

In Figure 12, despite the color modifications, an incorrect prediction is still observed, consistent with the original result, along with a similar attention distribution between Grad-CAM and Grad-CAM++, with a slightly more widespread activation compared to the original case for experiment 11 in Figure 12a. Using the experiment 12 model (Figure 12b), instead, the prediction is correct and the activation maps are consistent with the original ones. In Figure 12, for experiment 11 (Figure 12c), the Grad-CAM maps differ significantly from the original, showing stronger activation in the upper part of the image, corresponding to the ceiling. For experiment 12 in Figure 12d, instead, the prediction is correct and the activation maps are consistent with the original ones.

Overall, this ablation study suggests that clothing color does not significantly influence the model’s decisions when predictions are correct. However, in cases of misclassification, color modifications may affect both the attention regions highlighted by Grad-CAM and, in some cases, the final prediction outcome.

**Figure 13:**
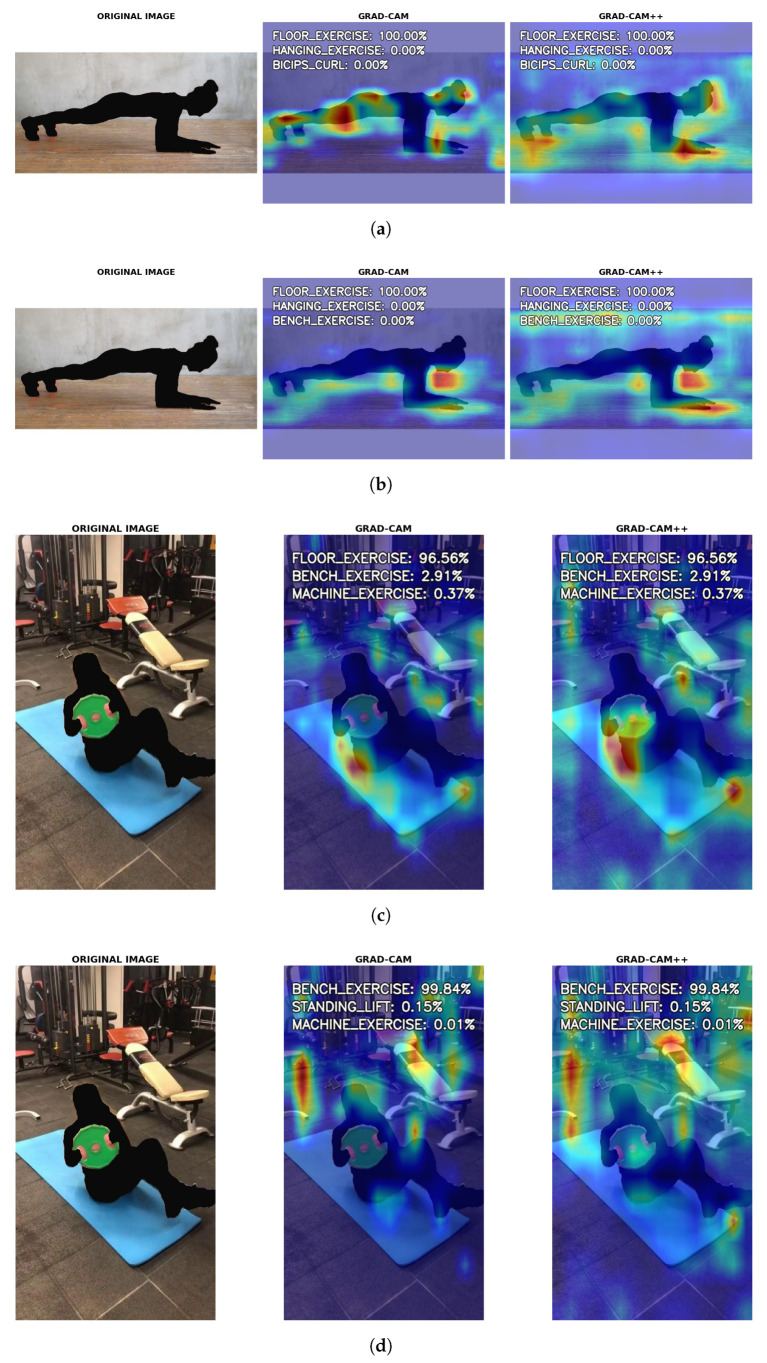
CAMs illustrating the model’s attention for the floor exercise class predictions with the mask applied to the person. (**a**) Experiment 11, plank exercise; (**b**) Experiment 12, plank exercise; (**c**) Experiment 11, russian twist exercise; (**d**) Experiment 12, russian twist exercise.

We conducted an additional ablation study by applying a mask over the entire human body. In Figure 13a,b, it can be observed that, even after completely removing the visual information related to the person, the predictions remain consistent with the original ones, and the Grad-CAM maps show stronger activation on the silhouette. Despite the masking, the body shape during exercise execution remains recognizable. A similar behavior is observed in Figure 13c,d, where the predictions and Grad-CAM maps for experiments 11 and 12 remain consistent with the original results.

**Figure 14:**
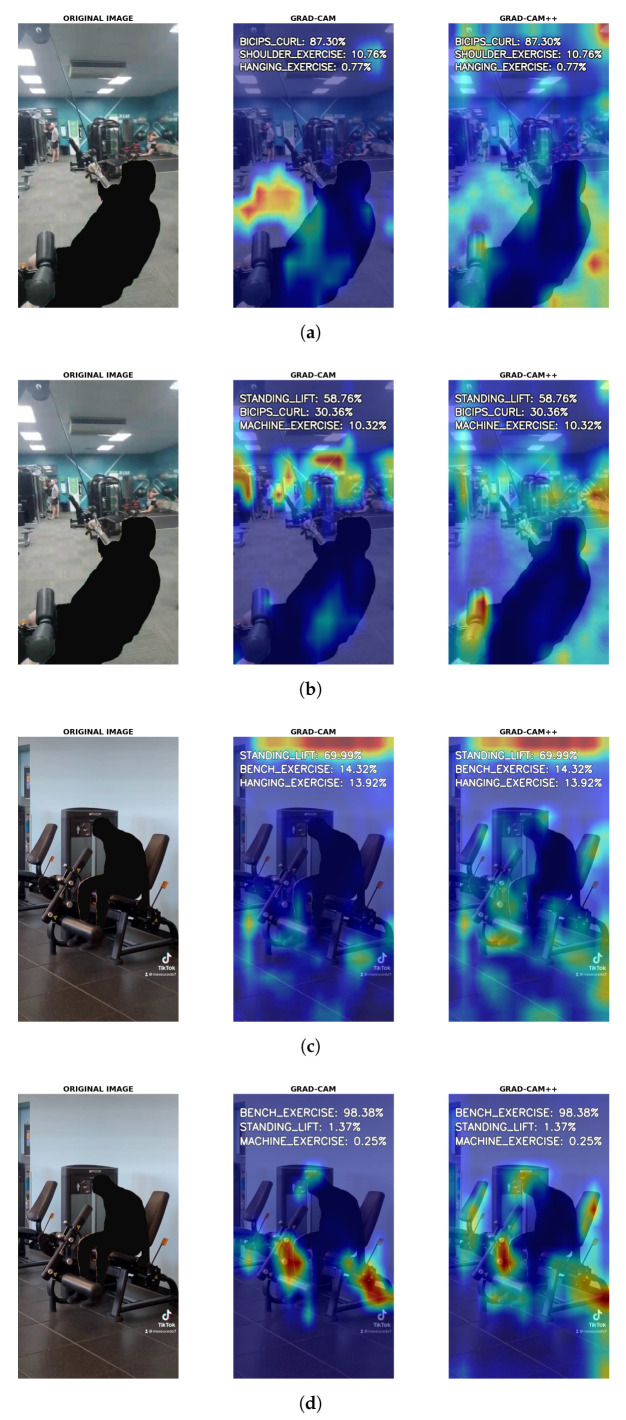
CAMs illustrating the model’s attention for the machine exercise class predictions with the mask applied to the person. (**a**) Experiment 11, lat pulldown exercise; (**b**) Experiment 12, lat pulldown exercise; (**c**) Experiment 11, leg extension exercise; (**d**) Experiment 12, leg extension exercise.

In contrast, in Figure 14a,b, applying the mask over the person leads to incorrect exercise classification. Similarly, in Figure 14c,d, obscuring the human figure results in changes in both predictions and Grad-CAM maps compared to the original images.

Overall, this second ablation study shows that the model from experiment 11, for the floor exercise class, is able to correctly recognize the exercise even when the person is masked. This suggests that, for bodyweight exercises, the silhouette alone may be sufficient for classification. On the other hand, the model from experiment 12 shows greater difficulty in recognizing the machine exercise class when the person is masked, compared to the original setting. This behavior may be due to the fact that exercises involving equipment require a holistic view that includes both the person and the apparatus used during the exercise execution.

## 5. Limitations

In this section, we discuss the limitations related to the proposed method. The proposed method is designed for single-user exercise monitoring, which aligns with the intended application scenario of individual training and personalized feedback. However, this setting does not consider multi-person environments, where multiple individuals may perform exercises simultaneously. Such scenarios represent an important direction for future work, particularly in real-world applications such as gyms, where robust person-level discrimination and separate attention mechanisms would be required.

## 6. Conclusions and Future Work

In this paper, we proposed a method for privacy-preserving, distributed workout video classification based on the YOLOv8 model, enriched with an explainability analysis that is performed through Grad-CAM and Grad-CAM++. The proposed method was evaluated on several exercises, reorganized by authors into eight biomechanically coherent categories, and trained across multiple heterogeneous clients in non-IID conditions. The proposed framework can be applied to several practical scenarios, including on-device fitness tracking, personalized workout feedback, home training safety, rehabilitation monitoring, and distributed gym environments. In these settings, multiple clients can collaboratively improve a shared model while preserving data privacy by avoiding the exchange of raw video data.

The experimental results suggest that the proposed approach is effective and stable across different federated configurations. In particular, the two best-performing setups, i.e., experiments 11 (FedAvg) and 12 (FedProx), show comparable performance, with training loss converging toward near-zero values (0.0005–0.002) and validation loss reaching 1.615 (FedAvg) and 1.605 (FedProx). The top-1 accuracy reached 0.675 for FedAvg and 0.672 for FedProx, while the top-5 accuracy showed a more pronounced difference, with FedAvg achieving 0.946 and FedProx reaching 0.980. These results indicate that both federated strategies are able to learn discriminative representations, and even when the top prediction is incorrect, the correct class is almost always included among the top five.

Explainability analyses highlights that Grad-CAM visualizations show that FedAvg often focuses on salient biomechanical cues but may be overly influenced by context when minority classes are involved, while FedProx tends to exhibit more stable and consistent attention to relevant exercise features and machine structures. Misclassifications revealed by CAMs suggest that contextual background elements (e.g., benches or gym machines) can influence predictions, underscoring the importance of explainability in diagnosing federated model behavior.

From the future work point of view, we plan to explore whether different versions of the YOLO model can help us to obtain better accuracy. Moreover, we will consider the integration of large language models with the aim of providing suggestions in case a gymnastic exercise is not correctly performed.

## Figures and Tables

**Table 1:** Distribution of images per class for each client across the training, validation, and test sets.

Client	Split	Floor	Bench	Standing	Biceps	Shoulder	Hanging	Machine	Tricep
Client 1	Train	1152	1359	1299	594	315	714	783	222
	Val	186	237	321	165	66	234	126	6
	Test	24	127	118	70	24	124	93	15
Client 2	Train	1362	867	1227	822	360	594	738	183
	Val	555	237	282	150	300	129	255	12
	Test	24	127	118	70	24	124	93	15
Client 3	Train	1200	1728	1341	510	444	918	885	165
	Val	210	75	165	132	96	555	474	84
	Test	24	127	118	70	24	124	93	15
Client 4	Train	963	1011	1176	519	351	435	570	150
	Val	759	141	186	168	222	342	66	12
	Test	24	127	118	70	24	124	93	15

**Table 2:** The hyperparameters for each experiment.

Exp	Total Clients	Epochs	Nr. Round	Aggregator	Batch Size	Learning Rate
1	2	8	3	FedAvg	16	0.01
2	2	8	3	FedProx	16	0.01
3	2	8	5	FedAvg	16	0.01
4	2	8	5	FedProx	16	0.01
5	3	8	3	FedAvg	16	0.01
6	3	8	3	FedProx	16	0.01
7	3	8	5	FedAvg	16	0.01
8	3	8	5	FedProx	16	0.01
9	4	8	3	FedAvg	16	0.01
10	4	8	3	FedProx	16	0.01
11	4	8	5	FedAvg	16	0.01
12	4	8	5	FedProx	16	0.01

**Table 3:** Results of each experiment per client, including training loss, validation loss, and top-1 and top-5 accuracies.

Exp	Client	Train Loss	Val Loss	Top-1 Acc	Top-5 Acc
1	1	0.010	1.770	0.497	0.945
	2	0.008	1.639	0.651	0.905
2	1	0.010	1.751	0.519	0.941
	2	0.008	1.691	0.589	0.900
3	1	0.004	1.795	0.473	0.922
	2	0.002	1.647	0.645	0.915
4	1	0.004	1.783	0.492	0.937
	2	0.002	1.645	0.65	0.919
5	1	0.010	1.785	0.480	0.940
	2	0.007	1.648	0.643	0.922
	3	0.008	1.615	0.663	0.975
6	1	0.009	1.793	0.478	0.926
	2	0.008	1.648	0.637	0.926
	3	0.008	1.632	0.642	0.972
7	1	0.004	1.781	0.484	0.920
	2	0.003	1.677	0.613	0.920
	3	0.003	1.643	0.637	0.974
8	1	0.004	1.748	0.518	0.950
	2	0.002	1.6877	0.598	0.910
	3	0.003	1.618	0.654	0.969
9	1	0.010	1.789	0.473	0.935
	2	0.007	1.688	0.591	0.914
	3	0.008	1.611	0.667	0.973
	4	0.022	1.7155	0.557	0.923
10	1	0.010	1.789	0.472	0.926
	2	0.008	1.661	0.629	0.921
	3	0.007	1.612	0.659	0.975
	4	0.010	1.757	0.513	0.904
11	1	0.004	1.758	0.516	0.928
	2	0.002	1.630	0.658	0.943
	3	0.003	1.644	0.624	0.971
	4	0.003	1.781	0.469	0.879
12	1	0.004	1.758	0.513	0.944
	2	0.003	1.664	0.630	0.918
	3	0.002	1.606	0.672	0.976
	4	0.003	1.814	0.436	0.887

**Table 4:** Distribution of images per class.

Split	Floor	Bench	Standing	Biceps	Shoulder	Hanging	Machine	Tricep
Train	4677	4965	5043	2445	1470	2661	2976	720
Val	1710	690	954	615	684	1260	921	114
Test	24	127	118	70	24	124	93	15

## Data Availability

The data presented in this study are available on request from the corresponding author.
